# Correlation Between Neutrophil to Lymphocyte Ratio and Myocardial Injury in Population Exposed to High Altitude

**DOI:** 10.3389/fcvm.2021.738817

**Published:** 2021-11-22

**Authors:** Siyi He, Shengdong He, Yongxiang Yang, Bin Li, Liang Gao, Qingyun Xie, Lin Zhang

**Affiliations:** ^1^General Hospital of Western Theater Command, Chengdu, China; ^2^Military Prevention and Control Center for Mountain Sickness, No. 950 Hospital of the Chinese People's Liberation Army, Yecheng, China

**Keywords:** neutrophil to lymphocyte ratio, high altitude, myocardial injury, myocardial enzymes, predictor

## Abstract

**Objective:** Myocardial injury is a severe complication in population exposed to high altitude. As a new biomarker for inflammatory response, neutrophil to lymphocyte ratio (NLR) has been widely used to predict the prognosis of various diseases. In this study, we intend to explore the risk factors for myocardial injury at high altitude and examine the relationship between NLR level and development of myocardial injury.

**Methods:** Consecutive patients admitted to a secondary general hospital at high altitude from June 2019 to May 2020 were selected into this retrospective study. Clinical and biochemical data were collected. According to the results of lactate dehydrogenase (LDH), creatine kinase (CK), creatine kinase isoenzymes (CK-MB), and aspartate amino transferase (AST), patients were divided into myocardial injury group and normal group.

**Results:** A total of 476 patients were enrolled in this study. Myocardial injury occurred in 158 patients (33.2%). We found that altitude, NLR, hemoglobin, total bilirubin, total cholesterol, and lipoprotein A in myocardial injury group were significantly higher than that in normal group (*P* < 0.05), while platelet count in myocardial injury group was significantly lower than that in normal group (*P* < 0.05). Logistic multivariate regression analysis revealed that there was an independent relationship between myocardial injury and smoke, NLR, hemoglobin (*P* < 0.05). By using Spearman correlation analysis, NLR was proved to have a significant positive correlation with LDH, CK, and CK-MB (*P* < 0.05) instead of AST. A receiver operating characteristic (ROC) curve was drawn to demonstrate that NLR could significantly predict the occurrence of myocardial injury with an area under the curve (AUC) of 0.594 (95% CI: 0.537–0.650, *P* < 0.05), and the level of 2.967 (sensitivity = 38.0%, specificity = 83.6%) was optimal cutoff value.

**Conclusion:** The incidence of myocardial injury is high in population at high altitude. Smoke, hemoglobin, and NLR are independent factors related to myocardial injury. As a convenient and efficient marker, NLR is found to be closely associated with myocardial enzymes and have a predict role in the occurrence of myocardial injury. This study will provide a theoretical basis on NLR for the early diagnosis of myocardial injury at high altitude.

## Introduction

High altitude could have a significant impact on physical health due to its specific environment. People exposed to high altitude mainly include residents, military force, and tourists who rush into the plateau. It has been long recognized that high altitude exposure is a type of cardiac stress and is associated with major changes in cardiovascular system (1, 2). Hypoxic and hypobaric environment will cause the damage of the cardiac cells, leading to the elevation of serum myocardial enzymes to varying degrees. Once myocardial injury occurs, the heart is less able to pump blood, which significantly affects individual ability to work and exercise at high altitude. In severe cases, malignant arrhythmia, heart failure, and even sudden death may appear (3). Therefore, more attention should be paid on the myocardial injury at high altitude.

Neutrophil to lymphocyte ratio is a widely studied indicator of inflammation in recent years. Multiple studies have demonstrated that neutrophil to lymphocyte ratio (NLR) can be used as a potential biomarker for predicting cardiovascular events and is closely associated with myocardial injury. In patients undergoing non-cardiac surgery, increased NLR level was found to be independently related to the development of myocardial injury (4). Cardiotoxicity is a common and severe complication once carbon monoxide poisoning, which could induce a significant upregulation in NLR level (5). Chen et al. conducted a single-center retrospective study in severe coronavirus disease 2019 (COVID-19) patients. It has been observed that NLR was an independent risk factor for myocardial injury and could predict myocardial injury with a sensitivity of 82.8% and a specificity of 69.5% (6).

In this study, young population exposed to high altitude were included. We intend to explore the risk factors for myocardial injury and examine the relationship between NLR level and development of myocardial injury, thereby providing theoretical basis for early clinical prevention and treatment.

## Materials and Methods

### Study Population

Consecutive patients admitted to a secondary general hospital at high altitude from June 2019 to May 2020 were selected into this retrospective study. Inclusion criteria were the following: (a) patients were all older than 18 years old; (b) all patients were exposed at an altitude of more than 3,000 km; (c) from entering the plateau to receiving hospital treatment, all patients did not leave the high-altitude environment. Exclusion criteria were the following: (a) there existed evidence of infection; (b) patients had a history of heart disease; (c) patients had a history of blood system disease; (d) patients were diagnosed with multiple organ dysfunction; (e) patients had a history of bleeding or blood transfusion within 1 week; (f) patients were previously exposed to high altitude for more than half a year; (g) clinical data were not collected completely.

### Data Collection and Laboratory Analysis

Basic information of patients was recorded, including age, sex, weight, ethnicity, altitude, exposure time at high altitude, smoking history, alcohol history, and so on. After admission, venous blood was urgently extracted from the patients, and myocardial enzyme, blood routine, liver function, kidney function, blood lipid, coagulation function, and other items were examined. Data were collected, such as lactate dehydrogenase (LDH), creatine kinase (CK), creatine kinase isoenzymes (CK-MB), aspartate amino transferase (AST), neutrophil count, lymphocyte count, hemoglobin, platelet count, red blood cell distribution width (RDW), total bilirubin, albumin, globulin, urea nitrogen, creatinine, uric acid, total cholesterol, triglyceride, high density lipoprotein cholesterin (HDL-C), low density lipoprotein cholesterin (LDL-C), lipoprotein A, prothrombin time (PT), activated partial thromboplastin time (APTT), D-dimer (D-D), and others.

### Patients Group

According to the results of myocardial enzymes, patients were included in the myocardial injury group when they met at least one of the following conditions: (a) LDH > 240 U/L; (b) CK > 200 U/L; (c) CK-MB > 25 U/L; (d) AST > 40 U/L. Patients with normal results of myocardial enzymes were included in the normal group. NLR was calculated based on the results of neutrophil count and lymphocyte count.

### Statistical Analysis

All analyses were performed by using the software SPSS version 23.0 (SPSS Inc., Chicago, IL, USA). Kolmogorov-Smirnov test was used to examine the normality of continuous variables. Measurement data of normal distribution were expressed as mean ± standard deviation, and the independent sample *t* test was used for comparison between groups. Measurement data of abnormal distribution were expressed as median (Q1–Q3), and Mann-Whitney U rank sum test was used for comparison between groups. Enumeration data were presented as *n* (%), and chi-square test was used for comparison between groups. Factors with *P* < 0.1 in univariate analysis were included in Logistic regression analysis as independent variables, while myocardial injury was regarded as the dependent variable. Odds ratio (OR) and 95% confidence interval (CI) were calculated. Spearman correlation analysis was estimated to determine the linear relationship between NLR and various myocardial enzymes. The diagnostic value of NLR in myocardial injury was assessed through the area under the curve (AUC) of the receiver operating characteristic (ROC) curve. The sensitivity, specificity, cutoff value, and accuracy were calculated correspondingly. Statistical difference was considered when *P* < 0.05.

## Results

### Baseline Characteristics of Included Patients

A total of 476 patients were enrolled in this study. Myocardial injury occurred in 158 patients (33.2%), including 21 cases with elevated LDH, 70 cases with elevated CK, 83 cases with elevated CK-MB, and 48 cases with elevated AST. The mean age of all patients was 24.6 ± 4.2 years, of whom 10 cases were female (2.1%). Included patients were exposed for 76.0 (30.5–112.0) days at altitude of 43.00 (3,900–5,200) m.

### Factors Related to Myocardial Injury in Population Exposed to High Altitude

As shown in Table 1, altitude [4,805.0 (4,100.0–5,200.0) m vs. 4,200.0 (3,900.0–5,175.8) m, *P* < 0.05], NLR [1.8 (1.3–4.5) vs. 1.6 (1.2–2.5), *P* < 0.05], hemoglobin [181.0 (163.8–208.0) g/L vs. 171.0 (159.3–185.0) g/L, *P* < 0.05], total bilirubin [16.9 (12.7–22.7) μmol/L vs. 15.1 (10.7–20.6) μmol/L, *P* < 0.05], total cholesterol [3.9 (3.2–4.3) mmol/L vs. 3.6 (3.0–4.1) mmol/L, *P* < 0.05], and lipoprotein A [146.0 (64.8–248.5) mg/L vs. 109.0 (59.0–198.3) mg/L, *P* < 0.05] in myocardial injury group was significantly higher than that in normal group, while platelet count [(205.9 ± 63.8) ^*^ 10^9^/L vs. (220.9 ± 60.6) ^*^ 10^9^/L, *P* < 0.05] in myocardial injury group was significantly lower than that in normal group. Compared with normal group, the percentage of smokers in myocardial injury group was higher (23.4 vs. 10.7%, *P* < 0.05). There was no significant difference between the two groups in age, sex, weight, nationality, exposure time at high altitude, alcohol, heart rate, RDW, albumin, globulin, urea nitrogen, creatinine, uric acid, triglycerides, HDL-C, LDL-C, PT, APTT, and D-D. In univariate analysis, there were 11 factors with *P* < 0.1, which were altitude, smoke, NLR, hemoglobin, platelet count, total bilirubin, urea nitrogen, uric acid, total cholesterol, LDL-C, and lipoprotein A. Logistic multivariate regression analysis was performed by taking the above 11 factors as independent variables, whereas taking the occurrence of myocardial injury as dependent variable. It was found that there was an independent relationship between myocardial injury and smoke (OR = 2.585, 95% CI: 1.020–6.552, *P* < 0.05), NLR (OR = 1.125, 95% CI: 1.007–1.256, *P* < 0.05), hemoglobin (OR = 1.011, 95% CI: 1.000–1.029, *P* < 0.05) (Table 2).

**Table 1:** Univariate analysis of factors related to myocardial injury in population at high altitude.

**Variables**	**Normal group (*n* = 318)**	**Myocardial injury group (*n* = 158)**	***P***
Age (year)	24.7 ± 4.3	24.4 ± 4.1	0.509
Female (*n*)	9	1	0.212
Weight (kg)	67.3 ± 8.8	67.9 ± 9.3	0.540
Han nationality	281	137	0.603
Altitude (km)	4,200.0 (3,900.0–5,175.8)	4,805.0 (4,100.0–5,200.0)	0.007^**^
Exposure days at high altitude	75.0 (32.3–109.5)	78.5 (30.0–112.3)	0.476
Smoke (*n*)	34	37	0.000^**^
Alcohol (*n*)	20	14	0.403
HR (min^−1^)	74.7 ± 11.8	73.9 ± 13.1	0.614
NLR	1.6 (1.2–2.5)	1.8 (1.3–4.5)	0.001^**^
Hemoglobin (g/L)	171.0 (159.3–185.0)	181.0 (163.8–208.0)	0.000^**^
Platelet count (*10^9^/L)	220.9 ± 60.6	205.9 ± 63.8	0.011^*^
RDW	41.0 (38.8–43.7)	41.5 (39.1–45.1)	0.142
Total bilirubin (μmol/L)	15.1 (10.7–20.6)	16.9 (12.7–22.7)	0.034^*^
Albumin (g/L)	43.0 (39.8–45.7)	43.9 (40.6–47.1)	0.278
Globulin (g/L)	26.4 (23.7–28.6)	26.4 (22.8–28.6)	0.279
Urea nitrogen (mmol/L)	5.5 (4.5–6.6)	5.7 (4.8–6.9)	0.060
Creatinine (μmol/L)	91.9 (82.4–109.8)	99.4 (82.1–121.3)	0.261
Uric acid (umol/L)	357.0 (237.5–433.0)	385.0 (268.5–466.5)	0.089
Total cholesterol (mmol/L)	3.6 (3.0–4.1)	3.9 (3.2–4.3)	0.013^*^
Triglyceride (mmol/L)	1.0 (0.7–1.4)	0.9 (0.7–1.4)	0.744
HDL-C (mmol/L)	1.1 (1.0–1.2)	1.1 (1.0–1.2)	0.596
LDL-C (mmol/L)	2.0 ± 0.6	2.2 ± 0.7	0.077
Lipoprotein A (mg/L)	109.0 (59.0–198.3)	146.0 (64.8–248.5)	0.045^*^
PT (s)	10.8 (10.1–11.9)	11.0 (10.3–12.4)	0.120
APTT (s)	29.9 (1.0–35.8)	30.5 (1.2–37.3)	0.104
D-D	0.2 (0.1–0.3)	0.2 (0.1–0.5)	0.713

*HR, heart rate; NLR, neutrophil to lymphocyte ratio; RDW, red blood cell distribution width; HDL-C, high density lipoprotein cholesterin; LDL-C, low density lipoprotein cholesterin; PT, prothrombin time; APTT, activated partial thromboplastin time; D-D, D-dimer. Bold values mean the comparison is statistically significant*.

* *Indicates significance at level 0.05*.

** *Indicates significance at level 0.01*.

**Table 2:** Logistic multivariate regression analysis of factors related to myocardial injury in population at high altitude.

	**B**	**SE**	**Wals**	**OR**	**95% CI**	***P***
Altitude	0.000	0.000	1.342	1.000	1.000–1.001	0.247
Smoke	0.950	0.475	4.004	2.585	1.020–6.552	0.045^*^
NLR	0.117	0.056	4.374	1.125	1.007–1.256	0.036^*^
Hemoglobin	0.011	0.006	3.838	1.011	1.000–1.029	0.048^*^
Platelet count	−0.005	0.003	3.653	0.995	0.990–1.000	0.056
Total bilirubin	0.014	0.011	1.474	1.014	0.992–1.036	0.225
Urea nitrogen	0.169	0.088	3.730	1.184	0.997–1.406	0.053
Uric acid	0.000	0.001	0.123	1.000	0.998–1.002	0.726
Total cholesterol	0.224	0.456	0.241	1.251	0.511–3.060	0.624
LDL-C	0.056	0.591	0.009	1.057	0.332–3.364	0.925
Lipoprotein A	0.001	0.001	1.236	1.001	0.999–1.003	0.266

*NLR, neutrophil to lymphocyte ratio; HDL-C, high density lipoprotein cholesterin. Bold values mean the comparison is statistically significant*.

* *Indicates significance at level 0.05*.

### Correlation Between NLR and Myocardial Injury Indexes

**Figure 1:**
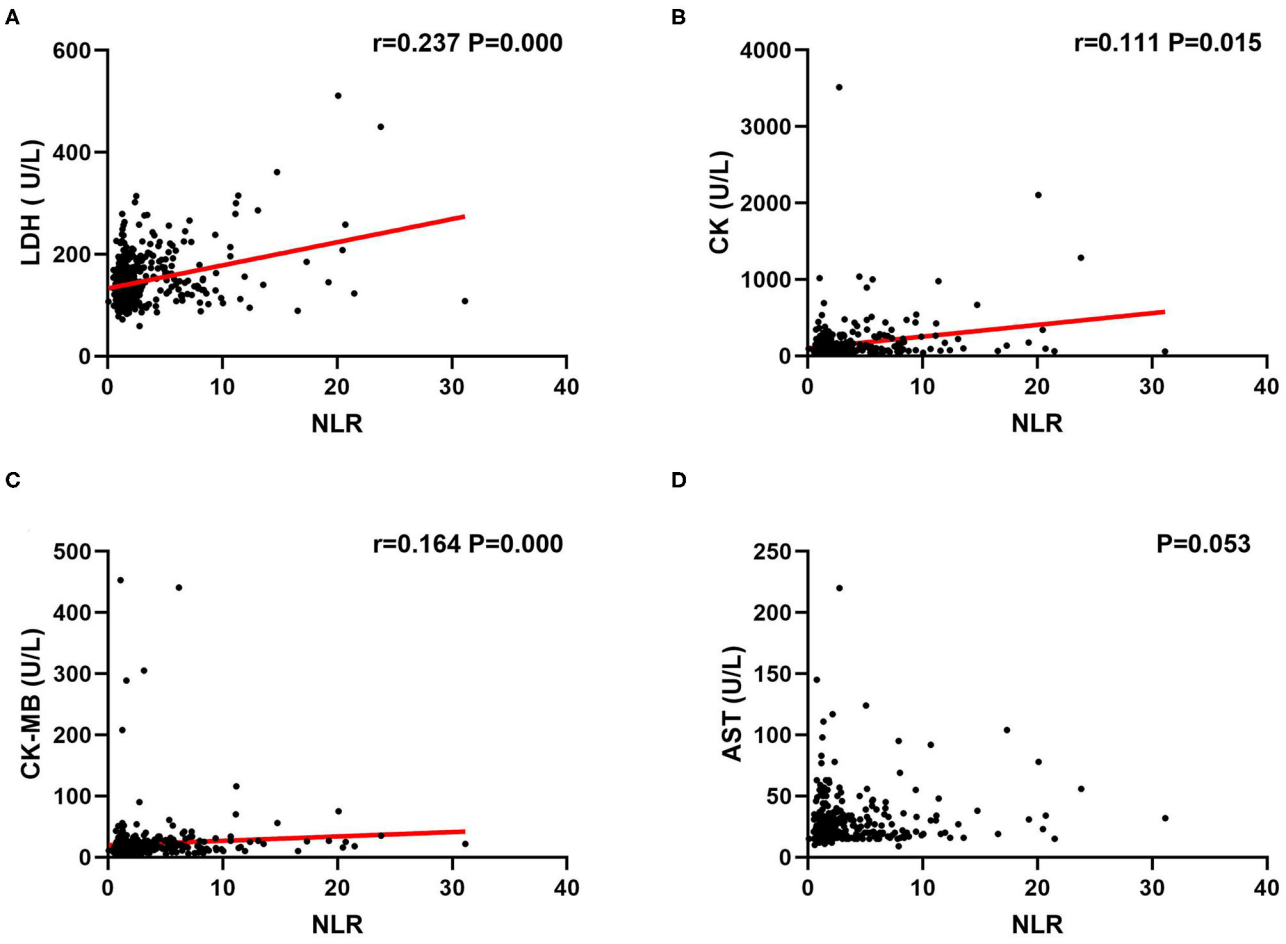
Correlation between NLR and myocardial enzymes at high altitude by Spearman correlation analysis. NLR had a significant positive relationship with LDH **(A)**, CK **(B)** and CK-MB **(C)** instead of AST **(D)**. Correlation coefficient (r) and *P* values are shown. NLR, neutrophil to lymphocyte ratio; LDH, lactate dehydrogenase; CK, creatine kinase; CK-MB, creatine kinase isoenzymes; AST, aspartate amino transferase.

Since NLR was found to be an independent factor related to myocardial injury, we further examined the association between NLR and myocardial injury indexes by using Spearman correlation analysis. Our results suggested that there was a relatively weak positive correlation between NLR and LDH (*r* = 0.237, *P* < 0.05, Figure 1A), CK (*r* = 0.111, *P* < 0.05, Figure 1B), CK-MB (*r* = 0.164, *P* < 0.05, Figure 1C) with significant difference, while AST had no significant correlation with NLR (*P* = 0.053, Figure 1D).

### Predictive Role of NLR in Myocardial Injury

**Figure 2:**
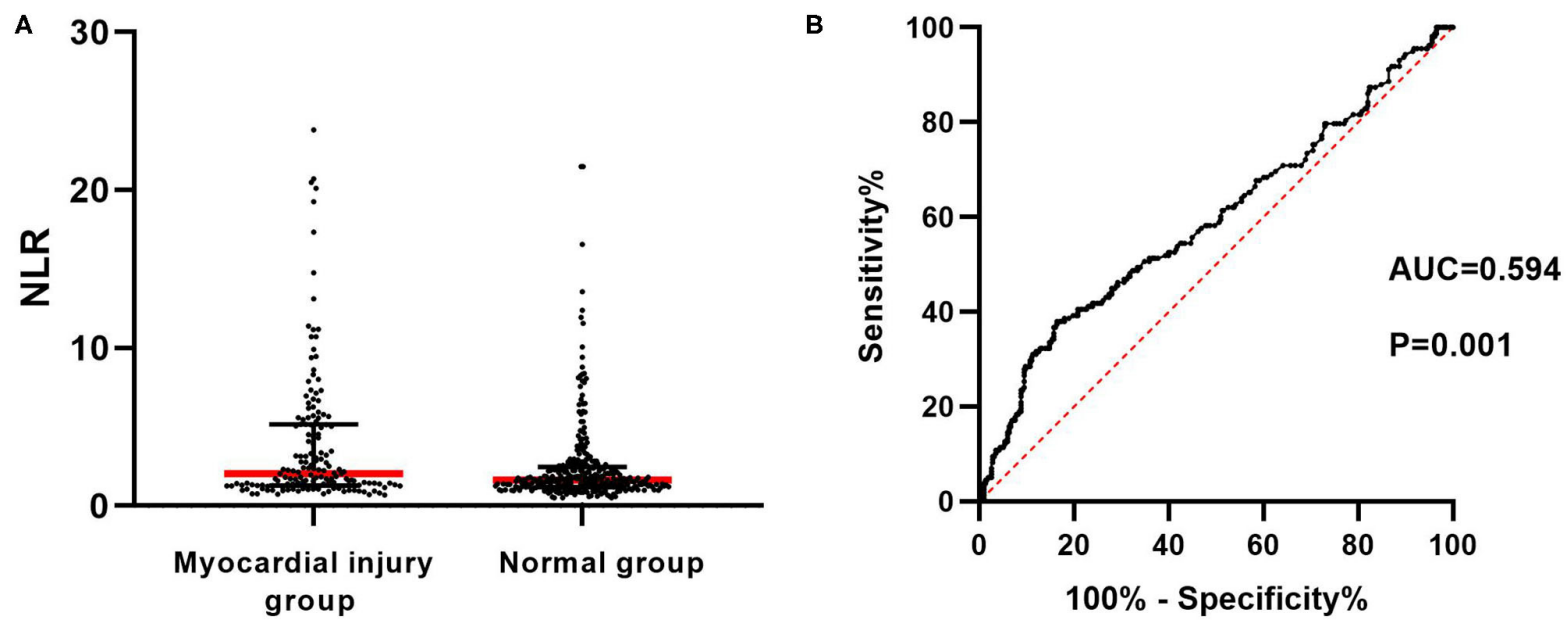
Predictive role of NLR in the occurrence of myocardial injury at high altitude. **(A)** Scatter plot of NLR values in myocardial injury group and normal group respectively. Red transverse line represents the median in each group. Black transverse line represents the upper and lower quartile in each group. **(B)** ROC curve revealed that NLR could significantly predict myocardial injury with AUC of 0.594. NLR, neutrophil to lymphocyte ratio; ROC, receiver operating characteristic; AUC, area under the curve.

The distribution of NLR in both myocardial injury group and normal group was shown as scatter plot in Figure 2A. As shown in Figure 2B, ROC curve was drawn to demonstrate that NLR level could predict the occurrence of myocardial injury with an AUC of 0.594 (95% CI: 0.537–0.650, *P* < 0.05) to a limited extent. An optimal cutoff value of 2.967 was determined using maximal Youden's index with sensitivity of 0.380 and specificity of 0.836.

## Discussion

The heart is highly sensitive to hypoxia. When population reaches the plateau, the cardiomyocytes are subjected to prolonged hypoxic stimulation. Hypoxia will result in the disorder of energy metabolism in cardiomyocytes, accompanied by the accumulation of oxygen free radicals, thus destroying membranous permeability (7). On the other hand, ventricular hypertrophy is also a distinguishing feature of high-altitude heart disease (8). Abnormal thickening of the myocardial fibers will make cardiomyocytes in a relatively ischemic state, which is also the possible mechanism of myocardial injury at high altitude. The destruction of cell membrane will cause the massive release of intracellular myocardial enzymes into the blood, which is the clinical manifestation of myocardial injury. In recent years, with the increase of population entering into the plateau, cardiogenic accident has become a common catastrophic event with high mortality in high altitude (9, 10). All the subjects in this study were young people. Even so, the incidence of myocardial injury at high altitude could still be as high as 33.2%, which is enough to show the severe influence of the plateau environment on cardiomyocytes.

At present, there are few studies focusing on myocardial injury at high altitude. In this retrospective research, we found that smoke, hemoglobin, and NLR were independent factors related to myocardial injury under high-altitude exposure. Smoke has been demonstrated to be an independent predictor for adverse cardiovascular events in the past (11). Even after revascularization in patients with complex coronary artery disease, smoke predicts poor clinical outcomes during 5-year follow-up (12). Besides, smoke directly contributes to pulmonary arterial remodeling through increased cell senescence (13), which will further exacerbate right heart dysfunction once exposed to high altitude. This is probably because smoke could induce vascular and systemic inflammatory response, aggravate coronary endothelial injury, and cause vasomotor dysfunction. Smoke-free policies are encouraged in order to decrease the risk of incident cardiovascular disease (14). Based on our findings, smoking cessation is also recommended for people entering into the plateau to maximize health benefits. Hemoglobin is an intracellular oxygen-binding heme protein which will also be quickly released into the blood when cardiomyocytes are damaged. The predictive value of hemoglobin in the severity of coronary artery stenosis has been reported in previous literature (15). In patients with myocardial infarction, hemoglobin carries important independent prognostic information of major adverse events and mortality (16, 17). In addition, in order to maintain normal activities in the hypoxic environment at high altitude, there will be a compensatory increase in hemoglobin level to meet the oxygen supply of the body (18, 19). However, excessive hemoglobin accumulation in the vessels will inevitably increase the blood viscosity, leading to microthrombus formation and microcirculation disturbance in the distal coronary arteries, thus further aggravating the myocardial injury. Besides that, we noticed that patients in myocardial group had higher lipoprotein A and total cholesterol by univariate analysis. High level of blood lipids could indeed increase the risk for cardiovascular events. Blockage of coronary microvessels would lead to local myocardial ischemia, which is also the possible mechanism of myocardial injury.

Neutrophils can secrete a variety of cytokines to regulate the function of cells, while lymphocytes play an important role in immune regulation. NLR systematically reflects the state of inflammatory response in the body and accurately predicts the prognosis of various diseases. For patients with congenital heart disease with pulmonary hypertension, our previous study demonstrated that NLR was closely related to early postoperative complications and could act as a novel predictor for prolonged mechanical ventilation time (20). In a recent study, NLR and other inflammatory markers were found to be elevated associated with hyperglycemia and larger infarct size in patients with acute myocardial infarction (21). Hypoxia could inevitably induce the activation of inflammatory cascade reactions, which is consistent with the degree of myocardial remodeling (22, 23). So, we speculate that NLR will be related to the myocardial injury under high altitude circumstance, which was exactly verified in this study. LDH, CK, CK-MB, and AST were detected to diagnosed myocardial injury. Although these four indicators are not as specific and sensitive as high-sensitivity troponin, they are still classic serum biomarkers of myocardial injury since the 1960s (24) and are widely used in quite a few clinical trials or animal experiments (25–27). Our findings suggested that NLR was closely associated with the expression of LDH, CK, CK-MB, and the level of 2.967 was the optimal cutoff value for predicting myocardial injury. Although AST is also a myocardial enzyme, it is widely present in multiple human tissues, such as red blood cells and hepatic cells. In particular, an increase in the number of red blood cells is usually concomitant with high altitude exposure, contributing to the upregulation of AST level once destroyed. Therefore, the specificity and sensitivity for the diagnosis of myocardial injury by AST are not high. In the plateau region, values of myocardial enzymes are not easy to achieve. As a common test data, blood routine has the advantages of being simple, fast, and cheap, from which NLR can be obtained. *Via* dynamically monitoring NLR, the inflammatory status and immune function of individuals could be accurately evaluated. NLR is helpful for prediction of myocardial injury at high altitude, and thus can provide early prevention and treatment strategies.

There still exist some limitations in the present research. Firstly, this study is a single-center retrospective study with limited number of included influencing factors. Secondly, all people exposed to high altitude were enrolled for analysis in our research. However, it was reported that chronic persistent hypoxia could induce compensatory adaptation of cardiomyocytes, and the extent of myocardial injury was lower than that of people with acute exposure to high altitude (28–30). Therefore, a prospective study in the future with distinction of subpopulation based on exposure duration is needed to obtain a higher-level proof of evidence-based medicine.

## Conclusion

In summary, incidence of myocardial injury is high in population at high altitude. Smoke, hemoglobin, and NLR are independent factors related to myocardial injury. As a convenient and efficient marker, NLR is found to be closely associated with myocardial enzymes and have a predicted role in the occurrence of myocardial injury. Our study will provide theoretical basis on NLR for the early diagnosis of myocardial injury at high altitude.

## Data Availability Statement

The raw data supporting the conclusions of this article will be made available by the authors, without undue reservation.

## Author Contributions

SiH, QX, and LZ conceptualized and designed the study. ShH, BL, and LG undertook data collection. SiH and YY performed statistical analysis. SiH wrote the manuscript. QX and LZ revised the manuscript. All authors contributed to the article and approved the submitted version.

## Funding

This study was granted by the 2021 annal project of the General Hospital of Western Theater Command (No. 2021-XZYG-B31).

## Conflict of Interest

The authors declare that the research was conducted in the absence of any commercial or financial relationships that could be construed as a potential conflict of interest.

## Publisher's Note

All claims expressed in this article are solely those of the authors and do not necessarily represent those of their affiliated organizations, or those of the publisher, the editors and the reviewers. Any product that may be evaluated in this article, or claim that may be made by its manufacturer, is not guaranteed or endorsed by the publisher.
